# Modification of Glassy Carbon Electrodes with Complexes of Manganese(II) with Some Phenanthroline Derivatives Immobilized in Nafion Layer

**DOI:** 10.3390/ijms25042348

**Published:** 2024-02-16

**Authors:** Danuta Tomczyk, Piotr Seliger

**Affiliations:** University of Lodz, Faculty of Chemistry, Department of Inorganic and Analytical Chemistry, Tamka 12, 91-403 Lodz, Poland; piotr.seliger@chemia.uni.lodz.pl

**Keywords:** manganese, phenanthroline, Nafion, modified electrodes, diffusion, electrocatalysis

## Abstract

Manganese(II) complexes with phenanthroline derivatives modified with different substituents were synthesized and incorporated into Nafion layers covering the surfaces of glassy carbon electrodes and were studied electrochemically. Formal potentials and apparent diffusion coefficients were calculated and discussed. The suitability for electrocatalytic oxidation of ascorbic acid and glycolic acid was examined. The surfaces of modified electrodes were characterized using atomic force microscopy.

## 1. Introduction

Mn ion complexes are one group of compounds that play an important role in catalysis [1,2,3,4]. They have found use as mediators of chemical reactions and act as selective receptors. For this reason, interest in the synthesis of Mn ion complexes to study their structural, chemical, electrochemical and oxidative properties has developed and continues to grow [5,6,7]. However, the problems of separating the catalyst from the reaction products and regenerating its active form are causing research to shift from homogeneous catalysis toward heterogeneous catalysis and electrocatalysis [8,9,10,11,12,13,14]. Catalysts are immobilized in inorganic materials [10] and in polymers [11,12]. This is a step toward increasing mediator stability and developing electrochemical sensors [14].

**Scheme 1:**
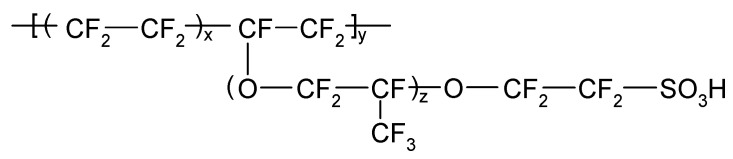
Nafion [23].

One of the methods used for the immobilization of electroactive complexes on an electrode surface for electrocatalytic purposes is incorporating them into a layer of an organic polymer [15,16,17,18,19]. One of them is a perfluorosulfonic polymer, commercially called Nafion^®^ (Scheme 1) (the registered trademark of E.I DuPont de Nemours), developed in 1962. Since then, it has found many applications [15,20,21] due to the ease of membrane preparation and its high chemical and thermal stability. Its chemical structure contains a hydrophobic perfluorocarbon backbone and hydrophilic perfluorosulfonic vinyl ether side chains. The hydrophilic segments form in Nafion membrane clusters where cationic substances can be firmly kept as the acid groups and can easily exchange hydrogen ions with other cations [22].

Charge transport through modified Nafion membranes can occur due to the physical diffusion of incorporated cations, electron hopping between redox centers or the movement of the supporting electrolyte [23,24]. The transport is influenced by the saturation of the membrane with a cation and its hydrophilicity. High saturation, especially with big hydrophobic complex cations, impedes the physical diffusion of the complex due to the dehydration of the membrane [25,26]. In such a case, however, there is still the possibility of an electron hopping from one reduced molecule to an adjacent oxidized one [27].

In our previous papers, we reported on the modification of a platinum electrode with a salen-type complex [28,29] and glassy carbon electrode surfaces with manganese complexes of unsubstituted 1,10-phenanthroline immobilized in a Nafion layer [30,31]. We examined their stability and the charge transport through the layer and calculated the apparent diffusion coefficients. These electrodes were used in electrocatalytic processes. In this paper, we extend the research to include some 1,10-phenanthroline derivatives in order to examine the influence of ligand substituent on electrode stability, diffusion coefficients and usefulness in electrooxidation processes (scheme of an example complex in ESI, Scheme S1).

The choice of ligands was motivated by the need to obtain manganese complexes that are as stable as possible (free Mn(III) ions in aqueous solution undergo disproportionation reactions in very significant amounts), are catalytically interesting and form cationic complexes. Phenanthroline and its derivatives are among the ligands that meet these criteria. The hard donor centers form chelate complexes with Mn(III) ions, a hard Lewis acid, and thus stabilize thermodynamically unstable ions. This is measured by the rather significant values of the formation constants [32] and data in the literature showing their stability [33,34,35]. Manganese complexes with phenanthroline show catalytic properties [36,37,38], giving hope to their application in heterogeneous electrocatalysis. In addition, they enable good binding to Nafion, which is caused by the proton exchange of the sulfonic groups of the cation-exchange polymer into positive complex ions.

Ascorbic acid and glycolic acid were chosen as reductants. The choice of ascorbic acid was driven by the very well-described mechanism of its catalytic oxidation in the literature [39,40], resulting from the very high interest in this compound. Ascorbic acid has been studied for a long time [39,40] and is still an object of catalytic research [41,42]. In the presence of various catalysts, the catalytic oxidation processes are stable, giving hope for a measurable electrocatalytic effect. The catalytic aspect of the second reductant is less studied, and the results we obtained can contribute to enriching these data.

## 2. Results and Discussion

### 2.1. Cyclic Voltammetry

The electrochemical activity and stability of the electrodes were tested through the use of long-term scanning in the potential range from 0.3 to 1.2 V vs. SCE in 0.1 M potassium nitrate aqueous solution. After an initial decrease, the peak currents became stable (maximally after 20 scans). This decrease may be due, first, to the swelling of dry Nafion caused by moisture [43]. It increases its volume and, as a consequence, decreases the concentration. This was confirmed by soaking the electrode in 0.1 M potassium nitrate water solution for ca. 20 min before the measurement, and then, the decrease was less distinct. Secondly, at the initial stage during the conditioning process, a series of processes forced by the electroneutrality requirement occur until equilibrium is reached. Then, the electroneutrality requirement during oxidation and reduction processes is ensured only by the flow of nitrate(V) anions.

**Figure 1:**
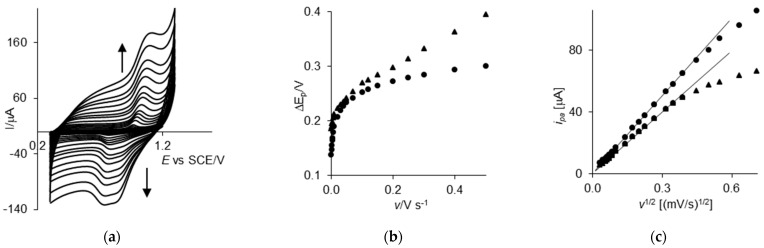
Cyclic voltammograms for [Mn(4–CH_3_–phen)_2_(H_2_O)_2_](ClO_4_)_2_ immobilized in Nafion layer on conventional GCE immersed in aqueous potassium nitrate (0.1 mol dm^−3^) recorded at different scan rates (v = 4, 5, 7, 10, 20, 30, 40, 50, 70, 100, 120, 150, 200, 250, 300, 400 and 500 mV s^−1^); 25 °C (**a**). Peak potential difference vs. scan rate ((**b**) ●) and anodic current vs. the root of scan rate ((**c**) ●) in comparison to the data obtained for [Mn(4–CH_3_–phen)_3_](ClO_4_)_2_ (▲); T = 295 K.

Figure 1 shows examples of the voltammetric curves recorded for [Mn(4-CH_3_-phen)_2_(H_2_O)_2_](ClO_4_)_2_ at scan rates ranging from 1 to 500 mV s^−1^. For all the redox couples under investigation, significant irreversibility is observed, and the peak potential difference (Δ*E_p_ = E_pa_* − *E_pc_*) changes with an increasing scan rate in the applied range from 140 to 380 mV (Figure 1b). Up to 20 mV s^−1^, the redox couples are quasi-reversible, and at higher rates, they become irreversible. For each of the ligands used, the recorded currents are higher for [MnL_2_]^2+^ than for [MnL_3_]^2+^ complexes (Figure 1c). In each case in plots *I_pa_ = f(v*^1/2^*)*, a linear interval is to be found, and this dependence is described in the Randles–Sevcik equation, which is valid for irreversible systems [44].

[MnL_2_]^2+^ complexes show a greater range of linearity, which may suggest higher kinetic constraints for [MnL_3_]^2+^ complexes.

Cyclic voltammetry measurements on the microelectrode were limited to low scan rates. Despite this fact, we did not reach the conditions of dominant spherical diffusion, which would be observed on voltammetric curves as constant current). At low scan rates (up to ca. 10 mV s^−1^), one can observe the comparable contribution of linear and spherical diffusion (mixed diffusion conditions).

**Figure 2:**
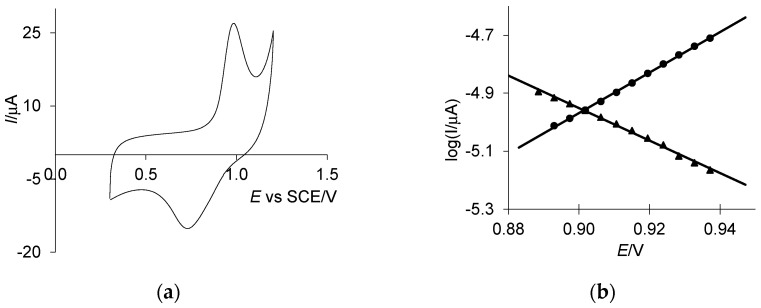
(**a**) Exemplary voltammetric curve recorded for [Mn(5–Cl–phen)_3_](ClO_4_)_2_ immobilized in Nafion layer on conventional GCE immersed in potassium nitrate (0.1 mol dm^−3^) at a scan rate of 20 mV s^−1^, T = 295 K, (**b**) and the polarization curves calculated on its basis.

Formal potentials were calculated based on the voltammetric curves recorded for scan rates where the redox couples were irreversible using the polarization curve method, which is suitable for such processes [45]. Exemplary voltammetric curves and polarization curves are presented in Figure 2a,b. In Table S1, ESI summarizes the calculated data for all the complexes under investigation.

The obtained *E*_f_^0^ values are lower than those found in the aqueous solutions of the corresponding complexes [32]. It reflects some interactions between the complexes and Nafion, and the difference in formal potentials may act as a measure of the strength of these interactions. They are strongest for complexes with nitro-derivative (with the formal potential lowering by 220 mV) and non-substituted phenanthroline (190 mV), and in the case of the other complexes, the lowering in values is 90–95 mV. The interactions are not very strong but distinct enough to bring additional stabilization to the thermodynamically unstable Mn(III) ions.

The transfer coefficients were calculated from equations valid for irreversible processes [45] using the voltammetric data recorded at scan rates where the linearity of dependence *I_pa_ = f(v*^1/2^*)* is kept. *E_pa_* − *E_pa/_*_2_ values were obtained using the analytical methods included in the AUTOLAB software package.

The calculated values of the anodic transfer coefficients are quite similar for different complexes, ranging from 0.47 to 0.54, and are slightly higher for [MnL_2_]^2+^ complexes, which is most likely due to the smaller steric effect caused by the smaller number of ligands.

The diffusion coefficients were calculated based on the Randles–Sevcik equation and data calculated earlier. The results are summarized in the Section 2.3.

#### Disproportionation Constants

The shape of the curves recorded for the complexes under investigation immobilized in the Nafion layer on the GC electrode surface is similar to those registered for aqueous solutions of the same objects with a pH ca. 3.0 [32]. The well-shaped peak observed in the anodic part is attributed to one-electron oxidation. The peak shifts toward higher potentials with an increasing scan rate (Figure 1a). The cathodic part shows a broadened peak, which, with increasing scan rates, shifts slightly towards negative potentials and changes its shape. The cathodic peak is lower than the anodic one, and the difference is greater the lower the scan rate is. This is typical behavior for an electrode process with the following disproportionation reaction:


$$2[\mathrm{Mn}L_{n}{]}^{2+}\overset{-2e}{⇌}2[\mathrm{Mn}L_{n}{]}^{3+}$$

$$2[\mathrm{Mn}L_{n}{]}^{3+}+2H_{2}O\overset{k_{1}}{⇌}[\mathrm{Mn}L_{n}{]}^{2+}+\mathrm{Mn}O_{2}+4H^{+}+\mathrm{nL}$$

The cathodic part of the voltammetric curve depicts the reduction of Mn(III) as well as MnO_2_. The reduction of MnO_2_ occurs at lower potential, and both processes partly overleap, which is observed as a broadening of the whole reduction peak. For free or weakly coordinated manganese ions, the manganese(IV) dioxide reduction peak is dominant. On the contrary, in the case of stable Mn(III) complexes, disproportionation equilibrium efficiently shifts left, and MnO_2_ reduction is observed only as a broadening of the Mn(III) reduction peak. Then, from the voltammetric measurements at different scan rates, one can calculate the rate constants of the disproportionation process following the electrode process [45]. For each scan rate, the ratios of anodic and cathodic peak currents were calculated, and for each of them, the kinetic parameter *ϖ* [44] was found, where *ϖ* is defined by the following equation:

(1) $$\log\varpi =\log(k_{1}c^{0}\tau )+0.047(a\tau -4)$$

where *aτ* is for processes where the oxidized form undergoes disproportionation:

(2) $$a\tau =\frac{nF}{RT}(E_{\lambda }-E_{f}^{0})$$

*c*^0^ represents the complex concentration in the layer, *k*_1_ represents the disproportionation constant, $E_{f}^{0}$ represents the formal potential, *E_λ_* represents the vertex potential, and *v* represents the potential scan rate.

The values of $k_{1}c^{0}\tau$ were calculated from Equation (1) and *k*_1_ values from the slope d$k_{1}c^{0}\tau$/d*τ*. The results are summarized in Table 1.

Disproportionation decreases the durability of the electrode and diminishes its usefulness in electrocatalysis. Taking this into account, complexes of lower *k*_1_ values would be preferred, which are generally observed for [MnL_3_]^2+^ complexes, which means that manganese(III) ions are better stabilized by three rather than two molecules of phenanthroline derivative ligands.

### 2.2. Chronocoulometry and Chronopotentiometry

**Figure 3:**
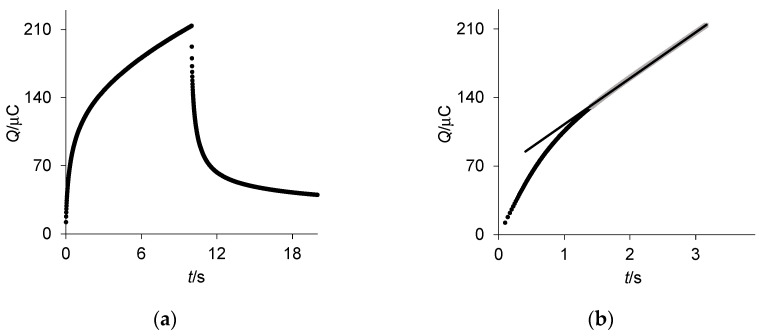
Exemplary chronocoulometric curve for conventional GCE modified with [Mn(4–CH_3_–phen)_2_(H_2_O)_2_](ClO_4_)_2_ immobilized in Nafion layer (**a**) recorded in 0.1 mol dm^−3^ aqueous potassium nitrate with corresponding *Q = f(t*^1/2^*)* relationship for the first step (**b**); T = 295 K.

In the case of chronocoulometric measurements registered on a conventional electrode, only linear diffusion takes place, even at higher pulse times, because the surface area of the electrode is much larger than the thickness of the layer. Then, the Cottrell equation [46] is valid, and from the slope of the relationship *Q = f(t*^1/2^*)*, the integrated Cottrell equation, known as the Anson equation, apparent diffusion coefficients could be calculated. Exemplary curves are presented in Figure 3a,b. The results are summarized in Table 2.

In the case of a microelectrode, the thickness of the layer (ca. 50 μm) is comparable to the electrode diameter, enabling the possibility of coulometric measurements under two time regimes. For a short pulse time (from 0.01 to 0.40 s), linear diffusion was dominant, and the Cottrell equation was used as described above for a conventional electrode. For time pulses longer than 0.40 s, additional spherical diffusion takes place. At the beginning of the measurement, the Cottrell reaction is valid, and the slope *Q/t*^1/2^ can be calculated. At the end of the same measurement, diffusion develops a spherical character, and the charge depends linearly on time:

(3) $$Q=4nFc^{0}Drt$$

And the *Q/t* value can be calculated directly from the slope of the terminal part of the chronocoulometric plot. From the system of Equations (4) and (5), one can obtain the diffusion coefficient and active complex concentration:

(4) $$D=\frac{{\left(Q/t\right)}^{2}\pi r^{2}}{4{\left(Q/t^{1/2}\right)}^{2}}$$

(5) $$c^{0}=\frac{{\left(Q/t^{1/2}\right)}^{2}}{nF\pi r^{3}(Q/t)}$$

The results are summarized in Table S2, ESI and Table 2. The calculated concentrations are in good agreement with the values estimated from the approximate layer density, although most of the values are lower than 3.8·10^−2^ mol dm^−3^, which may be caused by some loss of the complex during conditioning processes. Additionally, Nafion is an inhomogeneous medium, and a small percentage of complexes may stay entrapped in a region, excluding them from charge transport. Additionally, even at relatively long pulse times, diffusion does not have a purely spherical character, which can affect the accuracy of calculations.

The chronopotentiometric curves recorded for all the modified electrodes in 0.1 M potassium nitrate (Figure S1) show one-step oxidation of the complex immobilized in the Nafion layer. The curves are not well shaped and difficult to interpret, and all the data calculated on their basis may have only an approximate character. Nevertheless, it should be noted that at a constant complex concentration, the factor *Iτ*^1/2^ increases with increasing current (Table S3, ESI), confirming the chemical reaction following the electrode process, thus confirming the disproportionation of Mn(III) complexes.

The curves enable an evaluation of the anodic transfer coefficient (1−*α*)*n*_(__1−*α*)_ of the anodic form [45]. Despite the shape of the chronopotentiometric curves, the obtained values are in good agreement with those from the cyclic voltammetry measurements. The lack of fixed *Iτ*^1/2^ values makes chronopotentiometry unsuitable as another method for determining the diffusion coefficient.

### 2.3. Diffusion Coefficients Discussion

Table 2 below summarizes the diffusion coefficients received as described above. The precise estimation of diffusion coefficient values is difficult, even in homogenous solutions where an accuracy of 50% is considered good. Nafion has a complex, inhomogeneous and not quite well-known structure, so the accuracy must be even lower; hence, values of the same order, obtained through the use of four different methods, should be seen as satisfactory. The performed error calculation giving values of the order of 0.2 × 10^−8^–0.7 × 10^−8^ for *D_Red_* values from ~10^−8^ to ~5 × 10^−8^ mol cm^−2^ provides the basis for considering the diffusion coefficient values as verified.

Each of the methods has some disadvantages. Methods 1 through 3 rely on estimating the concentration of the complex in the layer, which does not take into account some loss of electroactive material during the conditioning process and is based on the density of unmodified Nafion. The 4th method avoids the problem and seems to be the most reliable despite the fact that, in this study, precise results were determined by achieving the conditions of dominant spherical diffusion, which was not always fulfilled.

Low accuracy makes it difficult to relate the diffusion coefficient values with other characteristics of the complexes or layers. Nevertheless, we can come to some qualitative conclusions. The order of *D_eff_* values indicates that charge transport is a result of complex ion diffusion rather than electron hopping (*D_eff_* values ranging from 10^−7^ to 10^−9^ mol cm^−2^ are attributed to physical diffusion [47]). Furthermore, complexes are stable in the Nafion layer, and the whole complex ions, not free manganese ions, undergo a diffusion process; otherwise, the *D_eff_* values would be an order higher, which could also be confirmed by the fact that diffusion coefficients for the [MnL_2_(H_2_O)_2_]^2+^ complexes are higher than those of the corresponding [MnL_3_]^2+^ complexes. Physical transport of the latter, which is larger and has a higher hydrophobicity, is more difficult in the relatively dense Nafion environment. Significantly low *D_eff_* values possess complexes with 5-nitophenanthroline, the most hydrophobic of the ligands under investigation. The highest values are shown by complexes with methyl substituent in ligand molecules despite the fact that the methyl group can cause steric hindrance, which can be explained by its more hydrophilic nature and different interactions with Nafion.

### 2.4. Electrocatalytic Activity against Reducing Agents

**Figure 4:**
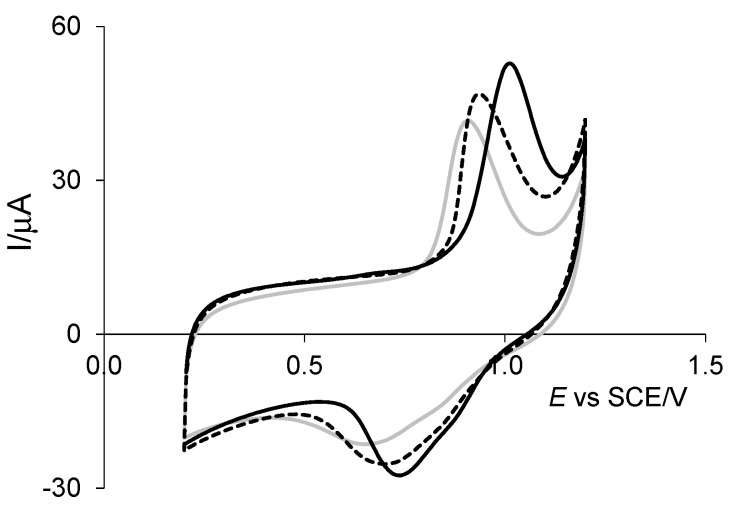
A comparison of chronovoltammetric curves recorded for an electrode modified with [Mn(4–CH_3_–phen)_2_(H_2_O)_2_](ClO_4_)_2_ immobilized in Nafion layer on GCE in potassium nitrate solution (grey line) and potassium nitrate with addition of ascorbic acid (black line) and glycolic acid (dashed line), scan rate 50 mV s^−1^, T = 295 K.

Electrocatalytic activity against ascorbic acid and glycolic acid was checked. None of them undergoes oxidation on the electrode modified with Nafion (in the absence of any manganese complexes). Voltammetric curves recorded for each modified electrode in 0.1 M potassium nitrate were compared with those recorded in aqueous solutions of the reductant (10^−3^ M) with potassium nitrate (0.1 M). Catalytic activity should be seen as an increase in the anodic peak, and an increase in anodic current was taken as a measure of catalytic activity. Table 3 presents the results. Example curves are shown in Figure 4 and Figures S2–S6.

Apart from the increase in the anodic peak, one can also observe other changes in the voltammetric curves: the anodic peaks shift towards more positive potentials as a result of weakening of the interactions between the complex and Nafion, and the cathodic peaks become narrower and higher in their frontal part, especially for [MnL_2_(H_2_O)_2_]^2+^ complexes, suggesting that the disproportionation product MnO_2_ also takes part in the oxidation of the reducing agent, Mn(III) ions are additionally stabilized by the reductant molecules and disproportionation constants are lower in such an environment.

Higher complex formation constants are advantageous in terms of the durability of the electrode; however, the presented data show that catalytic activity, taken as the relation of peak currents in the presence and absence of a reducing agent, changes in the opposite direction, and more stable complexes exhibit lower catalytic activity. The probable reason is the fact that the reductant used is involved in the simultaneous complexation of a manganese ion before electron exchange, which is easier the less stable the complex is. An additional argument in favor of this thesis is the shifting of the anodic peak potential towards more positive potentials in the presence of the reducing agent.

### 2.5. Microscopic Surface Characteristic

**Figure 5:**
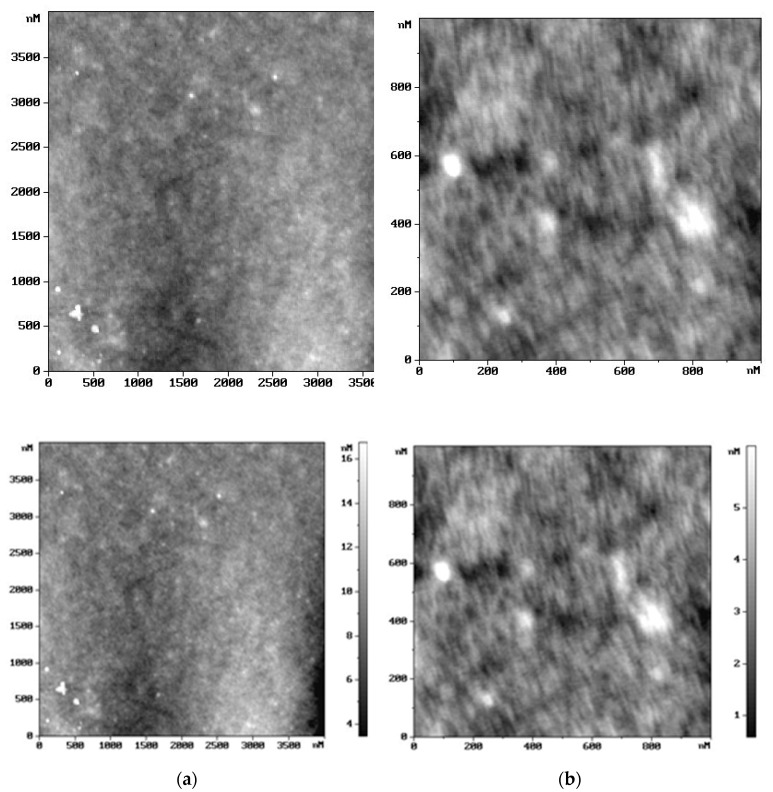
Surface profiles of a glassy carbon electrode covered with an unmodified Nafion layer at different scales: (**a**) 4000 × 4000 nM, (**b**)1000 × 1000 nM.

**Figure 6:**
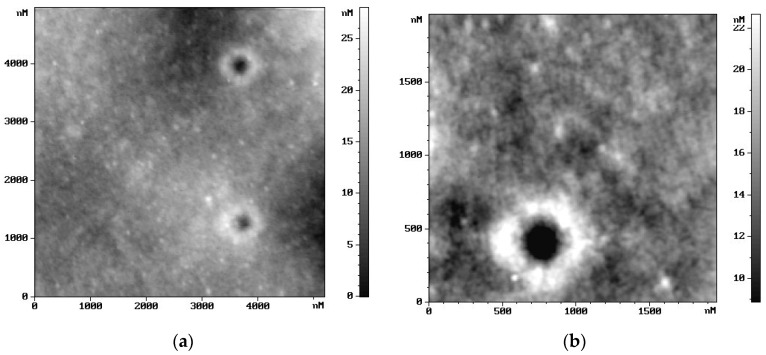
Surface profiles of a glassy carbon electrode covered with a Nafion layer modified with [Mn(5-CH_3_-phen)_3_](ClO_4_)_2_ after the drying process before any measurements at different scales: (**a**) 5000 × 5000 nM, (**b**) 2000 × 2000 nM.

**Figure 7:**
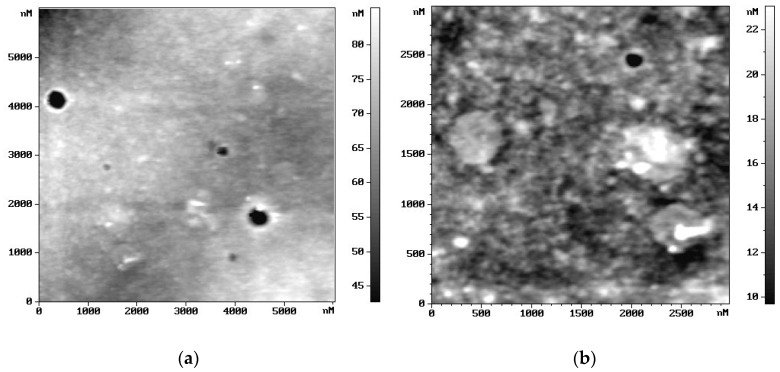
Surface profiles of a glassy carbon electrode covered with Nafion layer modified with [Mn(5-CH_3_-phen)_3_](ClO_4_)_2_ after the conditioning process (scanning in the potential range from 0.3 to 1.2 V vs. SCE in potassium nitrate aqueous solution, 0.1 mol dm^−3^) at different scales: (**a**) 6000 × 6000 nM, (**b**) 3000 × 3000 nM.

**Figure 8:**
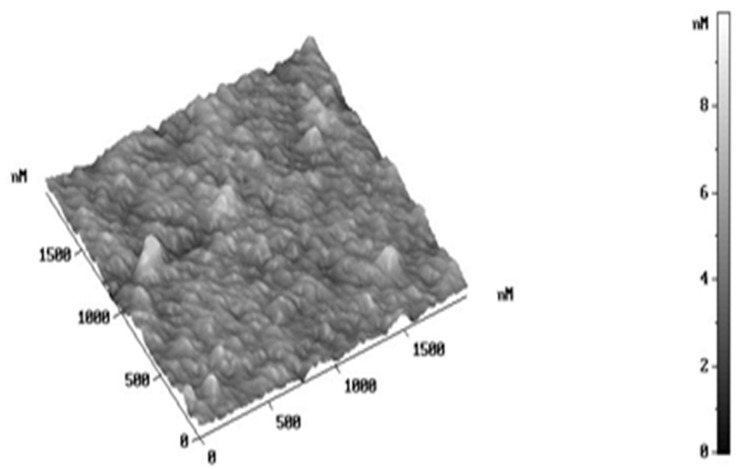
Topography of a glassy carbon electrode covered with a Nafion layer modified with [Mn(5-CH_3_-phen)_3_](ClO_4_)_2_ after the conditioning process (scanning in the potential range from 0.3 to 1.2 V vs. SCE in potassium nitrate aqueous solution, 0.1 mol dm^−3^).

The modified electrodes were scanned using atomic force microscopy (AFM). A series of measurements were made for the glassy carbon electrode modified with Nafion without any complexes (Figure 5), the electrode modified with Nafion with an immobilized complex directly after the drying process before any measurements (Figure 6), and finally, for the same electrode after the conditioning process via voltammetric scanning Figure 7. Figure 8 also presents an exemplary three-dimensional picture of one of the modifying layers.

Modifying layers possess a structurally heterogeneous topography (Figure 5). The distribution of the complex in the layer is distinctly heterogeneous, and in the picture, one can observe clusters as well as areas that are virtually empty (Figure 6). This observation is in agreement with studies on the structure of Nafion, which shows domains of extremely different hydrophilicity, influencing their affinity toward incorporated complex particles [48,49]. The comparison of the layers before (Figure 6) and after the conditioning process (Figure 7) shows some loss of material from the layer, but this is not important enough to explain the decrease in the observed peak, which points to other causes of the decrease.

## 3. Materials and Methods

### 3.1. Apparatus

Microanalyzer EDX Link 300 ISIS, Oxford Instruments, was used for energy-dispersive X-ray (EDX) analysis (determination of carbon, oxygen, chlorine and manganese).

All of the electrochemical measurements (cyclic voltammetry, chronocoulometry, chronopotentiometry) were carried out using the AUTOLAB (Eco Chemie) system in a three-electrode cell consisting of a modified glassy carbon electrode as the working electrode, a saturated calomel electrode (SCE) as the reference electrode and a perforated gold sheet as the auxiliary electrode.

Two glassy carbon electrodes (GCEs) were applied for modification: a conventional disc electrode (0.0685 ± 0.0007 cm^2^) and a microelectrode ((7.22 ± 0.08)·10^−4^ cm^2^). Their accurate sizes were determined earlier [30]. The GCE surfaces were conditioned by fine polishing before each coverage. No other pretreatments for the bare electrodes were carried out.

### 3.2. Reagents

Nafion^®^117 (~5% in a mixture of lower aliphatic alcohols and water) was purchased from Fluka, ligands (L), i.e., 1,10-phenanthroline (phen) and all the derivatives (4-methyl-, 5-methyl-, 5-chloro-, 5-nitro-, 4,7-dimethyl-, 5,6-dimethyl- and 2,9-dimethyl-1,10-phenanthroline) were obtained from Aldrich. Mn(ClO_4_)_2_⋅6H_2_O, glycolic acid and ethanol were obtained from Fluka. Ascorbic acid and Eriochrome Black T were obtained from POCh Gliwice (Gliwice, Poland). All of the reagents were used as received. Triple-distilled water was used throughout.

**Figure 9:**
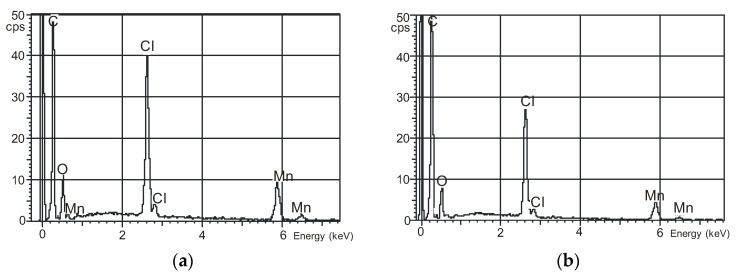
X-ray spectra of the complexes: [Mn(4-CH_3_-phen)_2_(H_2_O)_2_](ClO_4_)_2_ (**a**), [Mn(4-CH_3_-phen)_3_](ClO_4_)_2_ (**b**).

The solid complexes were synthesized and analyzed previously [30]. The chemical constitution of the complexes was confirmed using EDX analysis (exemplary spectra for [Mn(4-CH_3_-phen)_2_(H_2_O)_2_](ClO_4_)_2_ and [Mn(4-CH_3_-phen)_3_](ClO_4_)_2_ are presented in Figure 9), as well as through the use of colorimetry and complexometric titration of manganese(II) with EDTA in the presence of ascorbic acid as an antioxidant and Eriochrome Black T as an indicator. Similar to unsubstituted 1,10-phenanthroline, it was possible to receive complexes containing two or three ligand molecules in one complex ion, where, in the first case, two coordination sites are occupied by water molecules. The method of synthesis failed for 2,9-dimethyl-1,10-phenanthroline, which may be a result of very strong steric hindrance in the ligand molecule.

There are two ways to perform electrode modifications with a complex immobilized in a Nafion layer [50]. The first one, involving the preparation of a layer of Nafion and immersing the electrode in a solution of a complex to enter the polymer layer, was unsuitable in this case, as manganese–phenanthroline complexes are practically insoluble in water. One can obtain solutions of 1 mM in ethanol or mixtures of water/acetonitrile, but in these solvents, Nafion is partly soluble and does not adhere to the electrode material. This fact restricts the means of modifying the electrode surface to the second method, where the polymer and complex solutions are mixed previously and then put onto the surface of the electrode. We tested different proportions of Nafion and complex, and after initial examination for further research, we used the mixture that consisted of 3 parts by volume of the commercial Nafion solution and 7 parts by volume of the 1 mM ethanol complex solution. The resulting solution was poured onto the electrode surface (25 μL on conventional disc electrode and 3 μL on microelectrode). After the evaporation of well-adhering solvents, a transparent layer that completely covered the glassy carbon discs was formed. Higher complex concentration caused cracking in the layer, while lower ones gave indistinct responses on voltammograms. The concentration of complexes in the Nafion layer was evaluated at 3.8·10^−2^ mol dm^−3^, supporting the commonly taken density of wet Nafion 117 membrane, which is equal to 1.58 g cm^−3^ [51], on the assumption that the addition of the complex does not significantly influence the density of the ready film. The density reported later relating to the fully exchanged and water-treated Nafion film of ca. 1.90 g cm^−3^ [52] concerns less hydrophobic species than phenanthroline manganese complexes and the use of another method of film preparation, leading to the lower loading of the film. This assumption was verified experimentally (Section 0).

## 4. Conclusions

The incorporation of manganese complexes with phenanthroline derivatives into Nafion lowers the formal potentials of Mn(III)/Mn(II) coupling in comparison to similar systems in aqueous solutions, making manganese(III) ions more stable in a Nafion environment. The formal potentials, however, are still high enough to preserve the oxidizing properties of Mn(III), and such modified electrodes can be regarded as potential electrocatalysts. The stabilization of Mn(III) is better (because of the lower formal potentials) in complexes where all the coordination sites are occupied by phenanthroline-derivative ligands.

The calculated diffusion coefficients indicate that charge transport through the layer occurs due to the physical diffusion of complex ions. The rate of diffusion depends on the hydrophilicity of the complex and its interactions with Nafion. In general, complexes with two water molecules in their coordination sphere diffuse more easily than those with a coordination sphere saturated with phenanthroline-derivative molecules.

The suitability of modified electrodes for electrocatalytic oxidation depends on different factors. The 1:2 complexes are more easily transported through the membrane, and the redox centers seem to be more accessible to the reducing agent. However, 1:3 complexes are more stable, and the difference in catalytic effect, as compared to 1:2 species, is not significant. This suggests that complexes with coordination sites saturated with phenanthroline-derivative ligands are more convenient for electrocatalytic oxidation.

## Figures and Tables

**Table 1:** Disproportionation constants *k*_1_ of the oxidized forms of the complexes.

Complex	*k*_1_/dm^3^ mol^−1^ s^−1^	Complex	*k*_1_/dm^3^ mol^−1^ s^−1^
[Mn(4-CH_3_-phen)_2_(H_2_O)_2_]^3+^	41.6 ± 0.8	[Mn(4-CH_3_-phen)_3_]^3+^	21.7 ± 0.4
[Mn(5-CH_3_-phen)_2_(H_2_O)_2_]^3+^	52.5 ± 0.8	[Mn(5-CH_3_-phen)_3_]^3+^	34.8 ± 0.6
[Mn(5-Cl-phen)_2_(H_2_O)_2_]^3+^	20.5 ± 0.4	[Mn(5-Cl-phen)_3_]^3+^	4.6 ± 0.2
[Mn(5-NO_2_-phen)_2_(H_2_O)_2_]^3+^	4.6 ± 0.2	[Mn(5-NO_2_-phen)_3_]^3+^	7.3 ± 0.3
[Mn(4,7-CH_3_-phen)_2_(H_2_O)_2_]^3+^	61.2 ± 0.9	[Mn(4,7-CH_3_-phen)_3_]^3+^	54.8 ± 0.8
[Mn(5,6-CH_3_-phen)_2_(H_2_O)_2_]^3+^	112.7 ± 1.2	[Mn(5,6-CH_3_-phen)_3_]^3+^	33.3 ± 0.6

**Table 2:** Comparison of diffusion coefficients of manganese complexes with phenanthroline derivatives (*D_Red_*/mol cm^−2^) determined via cyclic voltammetry (cv) and chronocoulometry (cc) measurements recorded on a conventional electrode or on a microelectrode under linear or mixed diffusion conditions.

Complex	cv Conventional Electrode	cc Conventional Electrode	cc Microelectrode Linear Diffusion	cc Microelectrode Mixed Diffusion
[Mn(4-CH_3_-phen)_2_(H_2_O)_2_]^2+^	4.8 × 10^−8^	3.3 × 10^−8^	2.3 × 10^−8^	4.5 × 10^−8^
[Mn(4-CH3-phen)_3_]^2+^	4.1 × 10^−8^	2.4 × 10^−8^	5.7 × 10^−8^	4.4 × 10^−8^
[Mn(5-CH_3_-phen)_2_(H_2_O)_2_]^2+^	4.7 × 10^−8^	2.8 × 10^−8^	4.9 × 10^−8^	3.8 × 10^−8^
[Mn(5-CH3-phen)_3_]^2+^	4.0 × 10^−8^	2.7 × 10^−8^	5.9 × 10^−8^	2.6 × 10^−8^
[Mn(5-Cl-phen)_2_(H_2_O)_2_]^2+^	2.5 × 10^−8^	2.7 × 10^−8^	3.3 × 10^−8^	2.7 × 10^−8^
[Mn(5-Cl-phen)_3_]^2+^	1.7 × 10^−8^	3.8 × 10^−8^	3.0 × 10^−8^	2.3 × 10^−8^
[Mn(5-NO_2_-phen)_2_(H_2_O)_2_]^2+^	1.6 × 10^−8^	2.1 × 10^−8^	3.1 × 10^−8^	2.1 × 10^−8^
[Mn(5-NO_2_-phen)_3_]^2+^	0.6 × 10^−8^	3.0 × 10^−8^	3.0 × 10^−8^	2.6 × 10^−8^
[Mn(4,7-CH_3_-phen)_2_(H_2_O)_2_]^2+^	2.4 × 10^−8^	2.6 × 10^−8^	2.5 × 10^−8^	3.4 × 10^−8^
[Mn(4,7-CH3-phen)_3_]^2+^	2.2 × 10^−8^	2.4 × 10^−8^	2.8 × 10^−8^	3.1 × 10^−8^
[Mn(5,6-CH_3_-phen)_2_(H_2_O)_2_]^2+^	2.3 × 10^−8^	3.2 × 10^−8^	2.3 × 10^−8^	4.7 × 10^−8^
[Mn(5,6-CH_3_-phen)_3_]^2+^	2.1 × 10^−8^	4.1 × 10^−8^	2.1 × 10^−8^	4.1 × 10^−8^

Errors ranging from 0.2 × 10^−8^ to 0.7 × 10^−8^ mol cm^−2^ are omitted to simplify the table.

**Table 3:** The influence of the addition of different reductants on voltammetric responses of electrodes modified with different complexes immobilized in Nafion layer; scan rate, 50 mV s^−1^.

Complex	Increase in Anodic Peak High [%] in 10^−3^M Solution of Glycolic Acid Ascorbic Acid		Shift of Anodic Peak Potential [V] in 10^−3^M Solution of Glycolic Acid Ascorbic Acid	
[Mn(4-CH_3_-phen)_2_(H_2_O)_2_]^2+^	112	125	0.018	0.093
[Mn(4-CH_3_-phen)_3_]^2+^	116	139	0.025	0.081
[Mn(5-CH_3_-phen)_2_(H_2_O)_2_]^2+^	111	123	0.035	0.045
[Mn(5-CH_3_-phen)_3_]^2+^	112	129	0.018	0.044
[Mn(5-Cl-phen)_2_(H_2_O)_2_]^2+^	115	128	0.013	0.048
[Mn(5-Cl-phen)_3_]^2+^	120	164	0.045	0.040
[Mn(5-NO_2_-phen)_2_(H_2_O)_2_]^2+^	119	130	0.088	0.026
[Mn(5-NO_2_-phen)_3_]^2+^	123	173	0.004	0.013
[Mn(4,7-CH_3_-phen)_2_(H_2_O)_2_]^2+^	102	113	0.013	0.057
[Mn(4,7-CH_3_-phen)_3_]^2+^	106	131	0.027	0.044
[Mn(5,6-CH_3_-phen)_2_(H_2_O)_2_]^2+^	104	121	0.099	0.084
[Mn(5,6-CH_3_-phen)_3_]^2+^	108	134	0.034	0.045

## Data Availability

Data are contained within the article and Supplementary Material.

## Supplementary Materials

The following supporting information can be downloaded at: https://www.mdpi.com/article/10.3390/ijms25042348/s1.

## References

[1] Das K., Barman M.K., Maji B. (2021). Advancements in multifunctional manganese complexes for catalytic hydrogen transfer reactions. Chem. Commun..

[2] Friães S., Realista S., Gomes C.S.B., Martinho P.N., Royo B. (2021). Click-Derived Triazoles and Triazolylidenes of Manganese for Electrocatalytic Reduction of CO_2_. Molecules.

[3] Rohit K.R., Radhika S., Saranya S., Anilkumar G. (2020). Manganese-Catalysed Dehydrogenative Coupling—An Overview. Adv. Synth. Catal..

[4] Ballistreri F.P., Gangemi C.M.A., Pappalardo A., Tomaselli G.A., Toscano R.M., Sfrazzetto G.T. (2016). (Salen)Mn(III) Catalyzed Asymmetric Epoxidation Reactions by Hydrogen Peroxide in Water: A Green Protocol. Int. J. Mol. Sci..

[5] Papanikolaou M.G., Hadjithoma S., Gallos J.K., Miras H.N., Plakatouras J.C., Keramidas A.D., Kabanos T.A. (2021). Synthesis, structural and physicochemical properties of a series of manganese(II) complexes with a novel N5 tripodal-amidate ligand and their potential use as water oxidation catalysts. Polyhedron.

[6] Egekenze R.N., Gultneh Y., Butcher R. (2018). Mn(III) and Mn(II) complexes of tridentate Schiff base ligands; synthesis, characterization, structure, electrochemistry and catalytic activity. Inorg. Chim. Acta.

[7] Rigamonti L., Zardi P., Carlino S., Demartin F., Castellano C., Pigani L., Ponti A., Ferretti A.M., Pasini A. (2020). Selective Formation, Reactivity, Redox and Magnetic Properties of Mn^III^ and Fe^III^ Dinuclear Complexes with Shortened Salen-Type Schiff Base Ligands. Int. J. Mol. Sci..

[8] Wu Z., Wan T., Kong X., Shen Q., Li K., Wu H. (2023). Electrocatalytic hydrogen evolution at carbon paste electrodes doped with a manganese(II) imidazoledicarboxylate complex. Z. Naturforschung B.

[9] Wang M., Zhang S., Teng J., Zhao S., Li Z., Wu M. (2023). Combination of Mn-Mo Oxide Nanoparticles on Carbon Nanotubes through Nitrogen Doping to Catalyze Oxygen Reduction. Molecules.

[10] Sergienko N., Radjenovic J. (2021). Manganese oxide coated TiO_2_ nanotube-based electrode for efficient and selective electrocatalytic sulfide oxidation to colloidal sulphur. Appl. Catal. B Environ..

[11] Keypour H., Kouhdareh J., Karimi-Nami R., Alavinia S., Karakaya I., Babaei S., Maryamabadi A. (2023). Investigation of the electrocatalytic reaction for the oxidation of alcohols through the formation of a metal organic framework (Mn-MIL-100)/polymer matrix on the surface of an Au electrode. New J. Chem..

[12] Hefnawy M.A., Medany S.S., El-Sherif R.M., Fadlallah S.A. (2022). NiO-MnOx/Polyaniline/Graphite Electrodes for Urea Electrocatalysis: Synergetic Effect between Polymorphs of MnOx and NiO. ChemistrySelect.

[13] Soriano-López J., Elliott R., Kathalikkattil A.C., Ako A.M., Mulahmetović M., Venkatesan M., Schmitt W. (2020). Bioinspired Water Oxidation Using a Mn-Oxo Cluster Stabilized by Non-Innocent Organic Tyrosine Y161 and Plastoquinone Mimics. ACS Sustain. Chem. Eng..

[14] Peng R., Offenhäusser A., Ermolenko Y., Mourzina Y. (2020). Biomimetic sensor based on Mn(III) meso-tetra(N-methyl-4-pyridyl) porphyrin for non-enzymatic electrocatalytic determination of hydrogen peroxide and as an electrochemical transducer in oxidase biosensor for analysis of biological media. Sens. Actuators B Chem..

[15] Yang C., Gu Y., Zhang K.-L. (2023). Proton-Conductive and Electrochemical-Sensitive Sensing Behavior of a New Mn(II) Chain Coordination Polymer. Cryst. Growth Des..

[16] Chen T., Huang Z., Liu J., Jiang L., Chu J., Song C., Kong A. (2021). Mn-Pyridine N site-enriched Mn-N–C derived from covalent organic polymer for electrochemical oxygen reduction and capacitive storage. Ionics.

[17] Li H., Huang C., Li Y., Yang W., Liu F. (2019). Electrocatalytic reduction of trace nitrobenzene using a graphene-oxide@polymerized-manganese-porphyrin composite. RSC Adv..

[18] Saravanan N., Mayuri P., Huang S.-T., Kumar A.S. (2018). In-situ electrochemical immobilization of [Mn(bpy)_2_(H_2_O)_2_]^2+^ complex on MWCNT modified electrode and its electrocatalytic H_2_O_2_ oxidation and reduction reactions: A Mn-Pseudocatalase enzyme bio-mimicking electron-transfer functional model. J. Electroanal. Chem..

[19] Singh A., Hocking R.K., Chang S.L.-Y., George B.M., Fehr M., Lips K., Schnegg A., Spiccia L. (2013). Water Oxidation Catalysis by Nanoparticulate Manganese Oxide Thin Films: Probing the Effect of the Manganese Precursors. Chem. Mater..

[20] Gagliardi G.G., Ibrahim A., Borello D., El-Kharouf A. (2020). Composite Polymers Development and Application for Polymer Electrolyte Membrane Technologies—A Review. Molecules.

[21] Malara A., Bonaccorsi L., Fotia A., Antonucci P.L., Frontera P. (2023). Hybrid Fluoro-Based Polymers/Graphite Foil for H_2_/Natural Gas Separation. Materials.

[22] Seen A.J. (2001). Nafion: An excellent support for metal-complex catalysts. J. Mol. Catal. A Chem..

[23] Shoji M., Oyaizu K., Nishide H. (2008). Facilitated oxygen transport through a Nafion membrane containing cobaltporphyrin as a fixed oxygen carrier. Polymer.

[24] Peerce P.J., Bard A.J. (1980). Polymer films on electrodes. Part III. Digital simulation model for cyclic voltammetry of electroactive polymer film and electrochemistry of poly(vinylferrocene) on platinum. J. Electroanal. Chem..

[25] Rong D., Anson F.C. (1996). Severe inhibition of the diffusion and electroactivity of Cu(bpy)^2+^_2_ complexes incorporated in Nafion^®^ coatings on electrodes. J. Electroanal. Chem..

[26] Sun J., Jordan L.R., Forsyth M., MacFarlane D.R. (2001). Acid–Organic base swollen polymer membranes. Electrochim. Acta.

[27] Blauch D.N., Savéant J.-M. (1992). Dynamics of electron hopping in assemblies of redox centers. Percolation and diffusion. J. Am. Chem. Soc..

[28] Tomczyk D., Bukowski W., Bester K., Kaczmarek M. (2022). Electrocatalytic Properties of Ni(II) Schiff Base Complex Polymer Films. Materials.

[29] Tomczyk D., Seliger P., Bukowski W., Bester K. (2022). The Influence of Electrolyte Type on Kinetics of Redox Processes in the Polymer Films of Ni(II) Salen-Type Complexes. Molecules.

[30] Nowak L., Andrijewski G., Tomczyk D., Cichomski M. (2007). Modification of glassy carbon electrode with phenanthroline complexes of manganese(II) immobilized in nafion layer. Pol. J. Chem..

[31] Nowak L., Andrijewski G., Tomczyk D., Błaszczyk T. (2009). Transport of phenanthroline complexes of manganese(II) ions through nafion layer on glassy carbon electrode. Pol. J. Chem..

[32] Andrijewski G., Nowak L., Tomczyk D., Różański R., Dałkowski R. (2006). Complex formation equilibria and potentials of Mn(III)/Mn(II) couple in the presence of 1,10-phenanthroline derivatives in aqueous solutions. Pol. J. Chem..

[33] Zheng S., Gao L., Guo J. (2000). Synthesis and Characterization of Functionalized MCM-41 with Copper- and Manganese-Phenanthroline Complexes. J. Solid State Chem..

[34] Benton D.J., Moore P. (1973). Rates of formation and dissociation of complexes of manganese(II) with the ligands 1,10-phenanthroline, 2,2′-bipyridine, and 2,2′2″-terpyridine in anhydrous methanol. J. Chem. Soc. Dalton Trans..

[35] Ramalakshmi D., Reddy K.R., Padmavathy d., Rajasekharan M.V., Arulsamy N., Hodgson D.J. (1999). Polyiodides of transition metal trischelate cations: Syntheses, structures, and spectral and electrical conductivity studies of [Mn(phen)_3_](I_3_)_2_ and [Mn(bpy)_3_](I_3_)_1.5_(I_8_)_0.25_. Inorg. Chim. Acta.

[36] Goldsmith C.R., Jiang W. (2012). Alkene epoxidation catalyzed by manganese complexes with electronically modified 1,10-phenanthroline ligands. Inorg. Chim. Acta.

[37] Tikhonova L.P., Ivashchenko T.S. (1991). Study of bromate oxidation of complexes of manganese(II) with 1,10 phenanthroline. Theor. Exp. Chem..

[38] Zhang Y., Chen X., Zhang H., Ge X. (2022). Screening of catalytic oxygen reduction reaction activity of 2, 9-dihalo-1, 10-phenanthroline metal complexes: The role of transition metals and halogen substitution. J. Colloid Interface Sci..

[39] Hanaki A. (1969). Copper-catalyzed Oxidation of Ascorbic Acid. Chem. Pharm. Bull..

[40] Pournaghi-Azar M.H., Ojani R. (1995). Catalytic oxidation of ascorbic acid by some ferrocene derivative mediators at the glassy carbon electrode. Application to the voltammetric resolution of ascorbic acid and dopamine in the same sample. Talanta.

[41] Garakani S.S., Zhang M., Xie D., Sikdar A., Pang K., Yuan J. (2023). Facile Fabrication of Wood-Derived Porous Fe3C/Nitrogen-Doped Carbon Membrane for Colorimetric Sensing of Ascorbic Acid. Nanomaterials.

[42] Masihpour N., Hassaninejad-Darzi S.K., Sarvary A. (2023). Nickel-Cobalt Salen Organometallic Complexes Encapsulated in Mesoporous NaA Nanozeolite for Electrocatalytic Quantification of Ascorbic Acid and Paracetamol. J. Inorg. Organomet. Polym. Mater..

[43] Majsztrik P.W., Satterfield M.B., Bocarsly A.B., Benziger J.B. (2007). Water sorption, desorption and transport in Nafion membranes. J. Membr. Sci..

[44] Olmstead M.L., Nicholson R.S. (1969). Cyclic voltammetry theory for the disproportionation reaction and spherical diffusion. Anal. Chem..

[45] Galus Z. (1979). Electroanalytical Methods of Physical and Chemical Parameters Determination.

[46] Bard A.J., Faulkner L.R. (2001). Electrochemical Methods.

[47] Lyons M.E.G. (1990). Annual Reports on the Progress of Chemistry, Section C, Physical Chemistry.

[48] Yeager H.L., Steck A. (1981). Cation and water diffusion in nafion ion exchange membranes: Influence of polymer structure. J. Electrochem. Soc..

[49] Hsu W.Y., Gierke T.D. (1983). Ion transport and clustering in nafion perfluorinated membranes. J. Membr. Sci..

[50] Rubinstein I. (1985). The influence of the polymer structure on electrochemical properties of uncharged molecules in nafion films on electrodes. J. Electroanal. Chem. Interfacial Electrochem..

[51] Zook L.A., Leddy J. (1996). Density and Solubility of Nafion: Recast, Annealed, and Commercial Films. Anal. Chem..

[52] Oberbroeckling K.J., Dunwoody D.C., Minteer S.D., Leddy J. (2002). Density of nafion exchanged with transition metal complexes and tetramethyl ammonium, ferrous, and hydrogen ions: Commercial and recast films. Anal. Chem..

