# Supplementary material for: Modification of Glassy Carbon Electrodes with Complexes of Manganese(II) with Some Phenanthroline Derivatives Immobilized in Nafion Layer
Source: Int J Mol Sci. 2024 Feb 16;25(4):2348. doi: 10.3390/ijms25042348 (PMC10889230; doi:10.3390/ijms25042348)
Supplement: Supplementary file 1 [file ijms-25-02348-s001.zip › ijms-2862719-supplementary.pdf]

Supplementary materials

Modification of Glassy Carbon Electrodes with Complexes of Manganese(II) with some Phenanthroline Derivatives immobilised in Nafion layer

D. Tomczyk[*], P. Seliger

Scheme

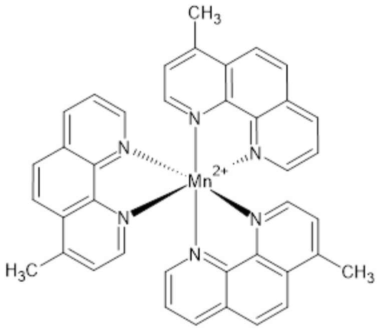

Scheme S1. Structure of $[Mn(4\text{-}CH_3\text{-phen})_3]^{2+}$.

Figures

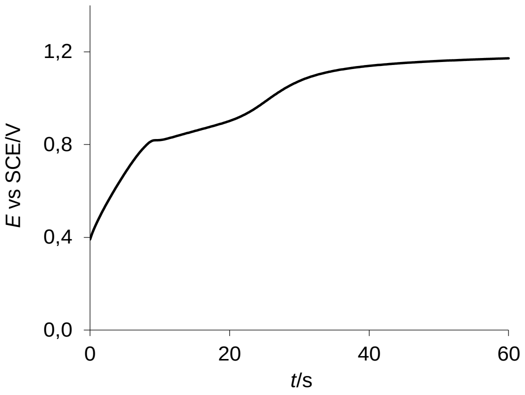

Figure S1. Chronopotentiometric curve for the conventional GCE modified with $[Mn(4\text{-}CH_3\text{-phen})_2(H_2O)_2](ClO_4)_2$ immobilized in Nafion layer recorded in 0.1 mol dm^{-3} potassum nitrate for $I = 10^{-5}$ A. T = 295 K.

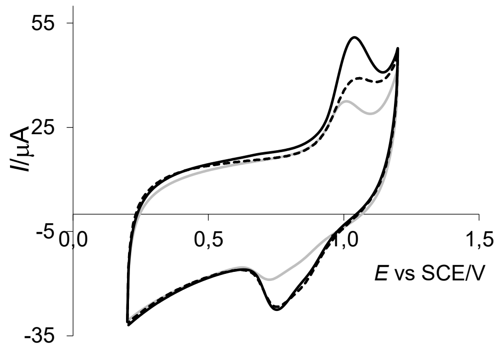

Figure S2. A comparison of voltammetric curves recorded for an electrode modified with [Mn(5-Cl_2-phen)$_3$](ClO_4)$_2$ immobilized in Nafion layer on GCE in potassium nitrate solution (grey line) and potassium nitrate with addition of ascorbic acid (black line) and glycolic acid (dashed line) , scan rate 50 mV s^{-1}, T = 295 K.

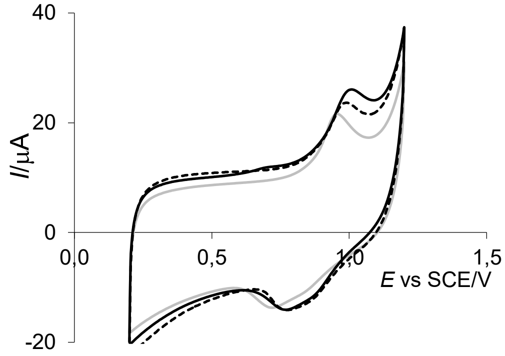

Figure S3. A comparison of voltammetric curves recorded for an electrode modified with [Mn(5-CH_3-phen)$_3$](ClO_4)$_2$ immobilized in Nafion layer on GCE in potassium nitrate solution (grey line) and potassium nitrate with addition of ascorbic acid (black line) and glycolic acid (dashed line) , scan rate 50 mV s^{-1}, T = 295 K.

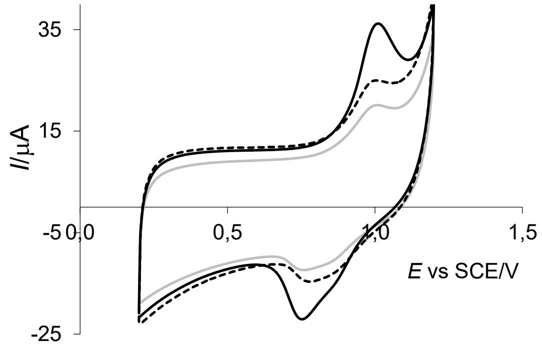

Figure S4. A comparison of voltammetric curves recorded for an electrode modified with [Mn(5-NO_2-phen)$_3$](ClO_4)$_2$ immobilized in Nafion layer on GCE in potassium nitrate solution (grey line) and potassium nitrate with addition of ascorbic acid (black line) and glycolic acid (dashed line) , scan rate 50 mV s^{-1}, T = 295 K.

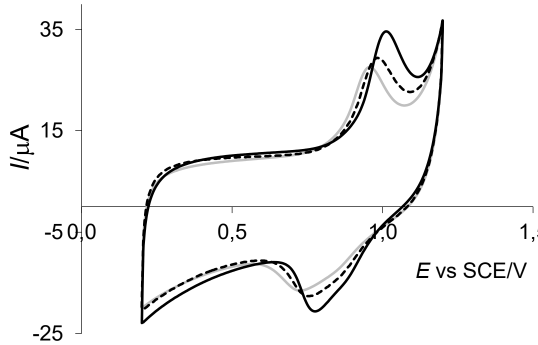

Figure S5. A comparison of voltammetric curves recorded for an electrode modified with [Mn(4,7-CH_3-phen)$_3$](ClO_4)$_2$ immobilized in Nafion layer on GCE in potassium nitrate solution (grey line) and potassium nitrate with addition of ascorbic acid (black line) and glycolic acid (dashed line) , scan rate 50 mV s^{-1}, T = 295 K.

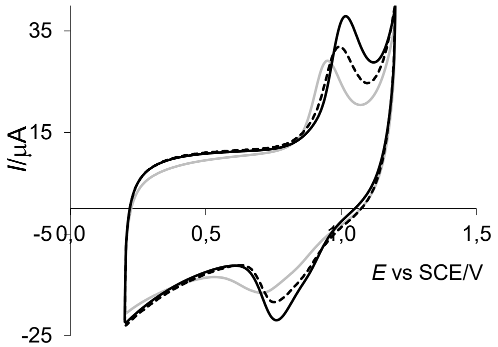

Figure S6. A comparison of voltammetric curves recorded for an electrode modified with [Mn(5,6-CH_3-phen)$_3$](ClO_4)$_2$ immobilized in Nafion layer on GCE in potassium nitrate solution (grey line) and potassium nitrate with addition of ascorbic acid (black line) and glycolic acid (dashed line) , scan rate 50 mV s^{-1}, T = 295 K.

Tables

Table S1. Formal potentials E_f^0 (*vs*. SCE) of Mn(III)/Mn(II) couple involved in complexes with phenanthroline and its derivatives immobilised in Nafion layer.

Complex	E_f^0/V	Complex	E_f^0/V
$[Mn(phen)_2(H_2O)_2](ClO_4)_2$ [a]	0,92±0.04	$[Mn(phen)_3](ClO_4)_2$ [a]	0,86±0.04
$[Mn(4\text{-}CH_3\text{-}phen)_2(H_2O)_2](ClO_4)_2$	0,90±0.04	$[Mn(4\text{-}CH_3\text{-}phen)_3](ClO_4)_2$	0,88±0.04
$[Mn(5\text{-}CH_3\text{-}phen)_2(H_2O)_2](ClO_4)_2$	0,89±0.04	$[Mn(5\text{-}CH_3\text{-}phen)_3](ClO_4)_2$	0,87±0.04
$[Mn(5\text{-}Cl\text{-}phen)_2(H_2O)_2](ClO_4)_2$	0,88±0.03	$[Mn(5\text{-}Cl\text{-}phen)_3](ClO_4)_2$	0,90±0.04
$[Mn(5\text{-}NO_2\text{-}phen)_2(H_2O)_2](ClO_4)_2$	0,90±0.04	$[Mn(5\text{-}NO_2\text{-}phen)_3](ClO_4)_2$	0,88±0.03
$[Mn(4,7\text{-}CH_3\text{-}phen)_2(H_2O)_2](ClO_4)_2$	0,89±0.04	$[Mn(4,7\text{-}CH_3\text{-}phen)_3](ClO_4)_2$	0,90±0.04
$[Mn(5,6\text{-}CH_3\text{-}phen)_2(H_2O)_2](ClO_4)_2$	0,87±0.03	$[Mn(5,6\text{-}CH_3\text{-}phen)_3](ClO_4)_2$	0,90±0.04

[a] data from [24]

Table S2. Complex concentrations calculated from chronocoulometric curves recorded on microelectrode in mixed diffusion conditions.

Complex	$c\cdot10^5$/mol cm^{-3}
$[Mn(phen)_2(H_2O)_2](ClO_4)_2$ [a]	3.8 ± 0.4
$[Mn(phen)_3](ClO_4)_2$ [a]	2.7 ± 0.3
$[Mn(4\text{-}CH_3\text{-}phen)_2(H_2O)_2](ClO_4)_2$	2.3 ± 0.3
$[Mn(4\text{-}CH_3\text{-}phen)_3](ClO_4)_2$	4.0 ± 0.5
$[Mn(5\text{-}CH_3\text{-}phen)_2(H_2O)_2](ClO_4)_2$	3.1 ± 0.4
$[Mn(5\text{-}CH_3\text{-}phen)_3](ClO_4)_2$	3.3 ± 0.4
$[Mn(5\text{-}Cl\text{-}phen)_2(H_2O)_2](ClO_4)_2$	2.2 ± 0.3
$[Mn(5\text{-}Cl\text{-}phen)_3](ClO_4)_2$	3.0 ± 0.4
$[Mn(5\text{-}NO_2\text{-}phen)_2(H_2O)_2](ClO_4)_2$	3.5 ± 0.4
$[Mn(5\text{-}NO_2\text{-}phen)_3](ClO_4)_2$	2.1 ± 0.3
$[Mn(4,7\text{-}CH_3\text{-}phen)_2(H_2O)_2](ClO_4)_2$	2.1 ± 0.3
$[Mn(4,7\text{-}CH_3\text{-}phen)_3](ClO_4)_2$	3.2 ± 0.4
$[Mn(5,6\text{-}CH_3\text{-}phen)_2(H_2O)_2](ClO_4)_2$	1.1 ± 0.4
$[Mn(5,6\text{-}CH_3\text{-}phen)_3](ClO_4)$	1.2 ± 0.4

[a] data from [25]

Table S3. The $I\tau^{1/2}$ dependence for $[Mn(4\text{-}CH_3\text{-}phen)_2(H_2O)_2](ClO_4)_2$ immobilized in Nafion layer calculated from potentiometric measurements.

I/A	τ/s	$I\tau^{1/2}$/A s$^{1/2}$
10^{-5}	13.5 ± 0,9	$3.7\cdot10^{-5}$
$2\cdot10^{-5}$	8.2 ± 0,7	$5.7\cdot10^{-5}$
$4\cdot10^{-5}$	4.1 ± 0,4	$8.0\cdot10^{-5}$
$8\cdot10^{-5}$	1.6 ± 0,2	$10.1\cdot10^{-5}$
10^{-4}	1.3 ± 0,1	$11.4\cdot10^{-5}$
